# Spinal hematoma after total knee arthroplasty: a case report

**DOI:** 10.1093/jscr/rjab354

**Published:** 2021-08-27

**Authors:** G Cortina, M Collarile, V Condello, R Orlandi, A Russo, V Madonna, N Pieracci

**Affiliations:** Department of Orthopaedic and Trauma Surgery, Campus Bio-Medico University, Rome, Italy; Department of Orthopedics, Joint Prosthetic, Arthroscopic Surgery and Sports Traumatology, Humanitas Castelli, Bergamo, Italy; Department of Orthopedics, Joint Prosthetic, Arthroscopic Surgery and Sports Traumatology, Humanitas Castelli, Bergamo, Italy; Department of Orthopedics, Joint Prosthetic, Arthroscopic Surgery and Sports Traumatology, Humanitas Castelli, Bergamo, Italy; Department of Orthopedics, Joint Prosthetic, Arthroscopic Surgery and Sports Traumatology, Humanitas Castelli, Bergamo, Italy; Department of Orthopedics, Joint Prosthetic, Arthroscopic Surgery and Sports Traumatology, Humanitas Castelli, Bergamo, Italy; Department of Neurosurgery, Humanitas Gavazzeni, Bergamo, Italy

**Keywords:** acute spinal hematoma, puncture-related complication, timely surgical decompression, rapid recovery

## Abstract

Spinal anesthesia is a common procedure performed in orthopedic surgery, and it is regarded as secure and safe. Although puncture-related complication of spinal anesthesia has a very low incidence, it would lead to dramatic neurological damage (tetra- or paraplegia). Early diagnosis and surgical decompression are mandatory to promote a better outcome. We present a case of acute spinal hematoma from T11 to L3, triggered by laborious anesthesia puncture after total knee arthroplasty. A prompt surgical decompression within few hours after diagnosis allowed rapid functional recovery and avoided permanent paraplegia.

## INTRODUCTION

Total knee arthroplasty (TKA) is one of the most-performed orthopedic procedures worldwide. The number of TKA per year is expected to rise by increasing the average age of the population [1]. In the setting of orthopedic surgery, spinal anesthesia is a standard procedure, and it is regarded as secure and safe [2]. A careful anamnestic evaluation of active or previous pathologies of the lumbar spine is usually performed before proceeding with a spinal anesthesia. Moreover, a radiographic examination of the lumbar spine is mandatory to ascertain any bone deformations (i.e. adult degenerative scoliosis) that can be misdiagnosed. In this case report, we present an acute spinal hematoma after TKA, which was triggered by a laborious spinal anesthesia. Furthermore, the purpose of this case report is to emphasize the role of early diagnosis and timely treatment in order to reach a better neurological outcome and to reduce permanent complications.

## CASE REPORT

A 77-year-old female was admitted in the orthopedics department to be operated for a TKA on her right knee. Her clinical history highlighted an ischemic stroke in 2019 without sequelae and hypertension. Preoperative workup, including coagulation profile and platelet count, was regular. The physical examination before surgery showed normal lower limb strength and function, no paresthesias and widespread pain in the right knee due to osteoarthritis with a range of motion (ROM), 5–125°.

**Figure 1:**
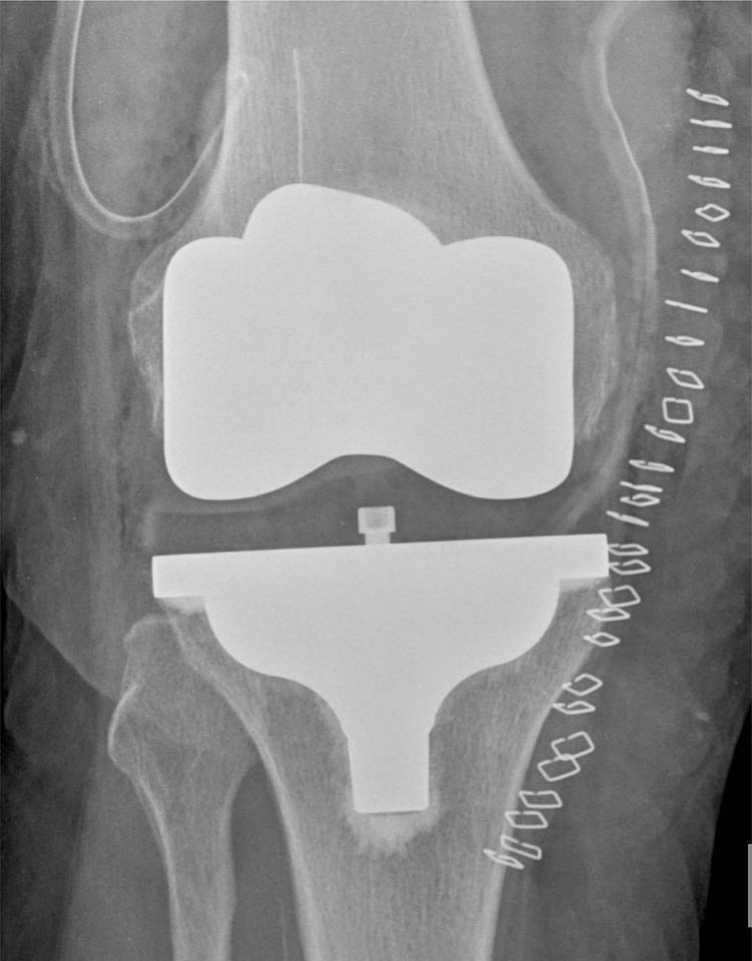
Post-operative TKA (Vanguard CR Zimmer Biomet, Warsaw, Indiana).

**Figure 2:**
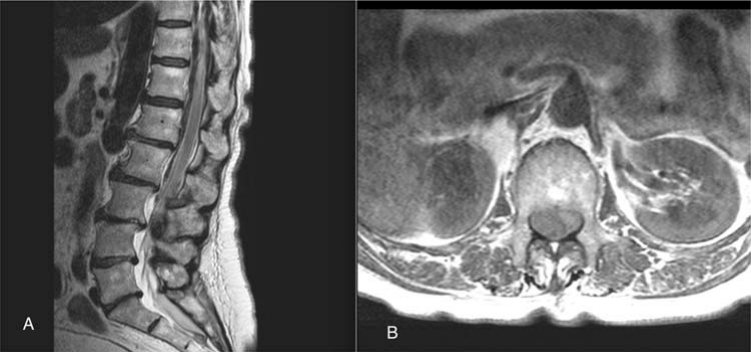
(**A**) The sagittal T2 FSE MRI show hypointense spindle-like mass from T10 to L3; (**B**) axial T1w FSE MRI showed iso-hyperintense hematoma compressing the dural sac (L1 level).

**Figure 3:**
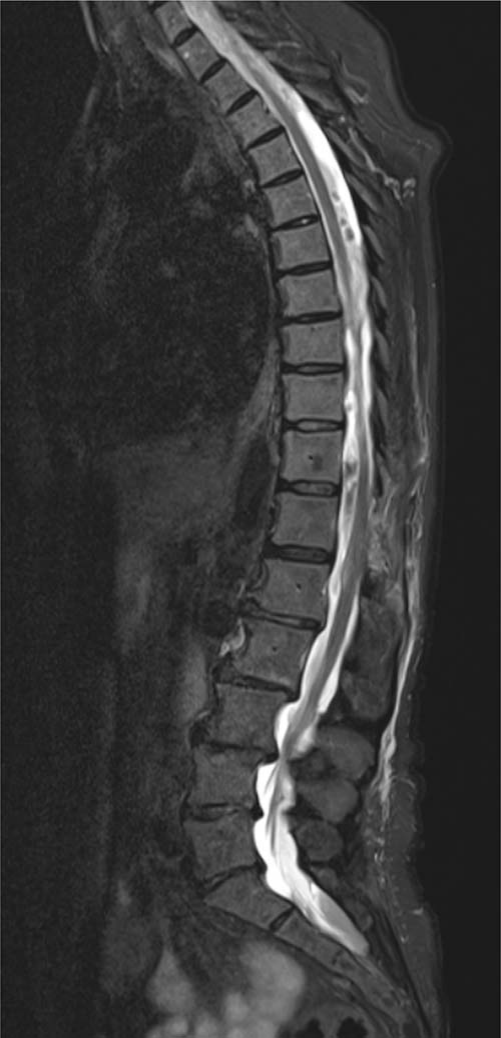
Follow-up MRI; it demonstrates a partial resolution of the hematoma with a confined area of myelopathy at the T12 level.

On 17 February 2020, she underwent TKA (Fig. 1). Spinal anesthesia was performed, with the patient in sitting position with one laborious puncture, as referred by the anesthesiologist. The patient received low molecular weight heparin (4000 UI enoxaparin sodium) subcutaneously 12 h after the surgery and a second shot dose 24 h later. On the second post-operative day (19 February 2020), early in the morning, the patient complained of numbness in her lower extremities and low back pain, partially relieved with analgesics. Four hours later, she was complaining for severe difficulty in moving her legs with increased low back pain. The physical examination showed flaccidly paralyzed left lower leg (0/5 left quadriceps, 0/5 left extensor digitorum longus, 0/5 left tibialis anterior and 0/5 left extensor hallucis longus), marked motor function reduction of the right lower leg (1/5 right quadriceps, 2/5 right extensor digitorum longus, 2/5 right tibialis anterior and 2/5 right extensor hallucis longus), sensory loss in both lower limbs, Babinski sign negative bilaterally and preserved sphincter functions. Magnetic resonance imaging (MRI) was required to rule out an acute spinal compression. The images showed a spindle-like mass, mostly intradural, from T11 to L3, hypointense in T2 and heterogeneous in T1 images compressing the dural sac (Fig. 2). Therefore, a diagnosis of acute spinal hematoma was made, and the patient was transferred to the Neurosurgery Department, and surgical decompression was scheduled. A bulky blood clot was removed through a spino-hemilaminectomy of T11 and T12 followed by a posterior dural incision. The source of bleeding was identified in a perimedullary vein on the dorsal surface of the spinal cord that was coagulated. Surgery was performed 3 h after diagnosis, and few hours later, the patient was able to perform a flexion-extension of both lower limbs. She was able to start walking with a walker 24 h later. A low molecular weight heparin (2000 UI Enoxaparin sodium) subcutaneously was started 1 week after surgical decompression. This dosage was increased to 4000 UI 2 days later. On 24 February 2020, a follow-up MRI was performed, and it showed a partial resolution of the hematoma with a confined area of myelopathy at the T12 level (Fig. 3). On the 26 February 2020, the patient started the rehabilitation protocol with the gradual improvement of the neurological status.

## DISCUSSION

Spinal hematoma is a rare complication of intraspinal anesthesia [3]. The incidence of spinal hematoma following intraspinal anesthesia is unknown but very low [4, 5]. In a retrospective study, Moen *et al*. has showed that epidural anesthesia has a higher risk than spinal anesthesia (1 in 18.000 vs. 1 in 158.000) [2]. Age (>70 years), thrombocytopenia or bleeding diathesis, such as renal or liver failure or anticoagulant therapy, are risk factors for spinal hematoma [6, 7]. Furthermore, in the orthopedic setting, aging may be an essential issue because it is linked with the prevalence of osteoporotic, arthritis degenerative changes and other pathologic processes of the spine that can make intraspinal anesthesia more laborious [8, 9, 10]. Generally, spinal anesthesia is usually performed at the L4–L5 or L3–L4 interspace, thus avoiding the possibility of injuring the spinal cord. In our case, the puncture was performed at the T11 level. Therefore, there was an evident mistake in the execution of the procedure since in this region, there are numerous radicular vessels, including the Adamkiewicz artery [11].

The clinical importance of this disorder is due to its sudden compressive effect on the spinal cord. The signs and symptoms are not different from other pathologies that can lead to acute compression of the spinal cord (tumor, trauma or vertebral abscess). In a patient with severe lumbar pain, acute or subacute, with a uni- or bilateral radicular pain, a spinal hematoma must be rule out [12]. If misdiagnosed, it may have catastrophic consequences. An early recognition is essential, and MRI is a sensitive imaging tool to confirm the suspicion. Findings on MRI depend on the timing of appearance of the clot: in the first 24 h, the hematoma is usually isointense on T1- and is hyperintense on T2-weighted images; after 24 h, it becomes hyperintense on T1 and on T2 [13].

This case report highlights the need to promptly recognize alarming signs and symptoms in order to proceed to a fast surgical decompression in case of spinal emathoma. It would reduce the risk of permanent neurological sequelae. In our case report, only about 3 h have elapsed since the diagnosis.

A better neurologic recovery, as seen in this case report, is more likely to occur if decompressive laminectomy is performed within 8 h of symptom onset than if surgery was over 24 h after symptom onset [14].
